# Author’s reply to “The nerve of blaming the curve”

**DOI:** 10.1007/s10151-021-02418-7

**Published:** 2021-02-16

**Authors:** S. E. van Oostendorp, R. Hompes, J. B. Tuynman

**Affiliations:** 1 Department of Surgery, Amsterdam UMC, Location VUmc, Cancer Center Amsterdam, Amsterdam, The Netherlands; 2 Department of surgery, Amsterdam UMC, location AMC, Cancer Center Amsterdam, Amsterdam, the Netherlands

Dear Sir,

We thank Dr. Orangio, Dr. Bergamaschi and colleagues for their comments on our article “Transperineal minimally invasive APE: preliminary outcomes in a multicenter cohort” [1, 2]. It is true that the transanal minimally invasive surgery (TAMIS) approach to an (extralevator) abdominoperineal excision is a new surgical concept and requires detailed assessment to judge its real impact and potential clinical benefit. This cohort study describes the first exploration of a new minimal invasive technique developed to improve outcomes of an extralevator abdominal perineal excision (ELAPE), by centers experienced in transanal minimal invasive procedures [1].

New surgical techniques should be published with honest reports of outcomes and if shown to have potential, further thorough assessment is necessary according to the Idea, Development, Exploration, Assessment, Long-term Follow-up, Improving the Quality of Research in Surgery (IDEAL) framework [3]. This cohort is the first report and is to be followed by properly designed prospective trials. The authors are well aware of these principles and we do not encourage implementation of new difficult techniques without the stages proposed in the IDEAL framework. Nevertheless, no improvement will be possible without an exploration phase which is presented in this cohort study.

ELAPE is a procedure which is associated with relatively poor short-term outcomes: wrong plane surgery, perineal wound infection and dehiscence as well as suboptimal oncological outcomes such as non-radical resection margins and local recurrence [4–6]. The TAMIS approach offers the potential to have better visualization of correct oncologic planes and does not require a large perineal skin incision. In our cohort, some of the advantages were observed such as low postoperative major morbidity and perineal dehiscence. But equally important, difficulties were encountered. Therefore, a timely honest report of the encountered benefits and risks was considered accommodating to the colorectal community.

This was indeed a cohort of locally advanced tumors situated in the low rectum. Considering the suboptimal histopathological outcomes, especially in the subgroup of unilateral ELAPE, it seems that a tailored ELAPE only excising the levator muscle at the invaded side is dangerous and the original Miles’ principles should be considered more appropriate; hence our call for more standardization upon continuation and subsequent evaluation of this procedure [7].

As technical advancements and surgical innovations by means of minimally invasive surgery enter daily practice, some surgeons may see benefits and potential areas for improvement and guide others to prevent them from making the same mistakes whereas others remain more conservative and antagonize new concepts repeatedly [8–10].

This is the first phase of exploration and honest report of benefits and difficulties. We do advise transparent reporting on new innovations. We do not advise the global surgical community to embark on this new technique at current stage, and encourage surgeons to further assess the efficacy and oncological safety according to the IDEAL framework.

On behalf of all our co-authors,

SE van Oostendorp,

R Hompes,

JB Tuynman.
